# Turbulent dispersal promotes species coexistence

**DOI:** 10.1111/j.1461-0248.2009.01427.x

**Published:** 2010-03

**Authors:** Heather A Berkley, Bruce E Kendall, Satoshi Mitarai, David A Siegel

**Affiliations:** 1 Donald Bren School of Environmental Science and Management, University of CaliforniaSanta Barbara, Santa Barbara, CA 93106-5131, USA; 2 Institute for Computational Earth System Science, University of CaliforniaSanta Barbara, Santa Barbara, CA 93106-3060, USA

**Keywords:** Community dynamics, fluid dynamics, larval dispersal, spatial storage effect, stable coexistence mechanisms, stochasticity

## Abstract

Several recent advances in coexistence theory emphasize the importance of space and dispersal, but focus on average dispersal rates and require spatial heterogeneity, spatio-temporal variability or dispersal-competition tradeoffs to allow coexistence. We analyse a model with stochastic juvenile dispersal (driven by turbulent flow in the coastal ocean) and show that a low-productivity species can coexist with a high-productivity species by having dispersal patterns sufficiently uncorrelated from those of its competitor, even though, on average, dispersal statistics are identical and subsequent demography and competition is spatially homogeneous. This produces a spatial storage effect, with an ephemeral partitioning of a ‘spatial niche’, and is the first demonstration of a physical mechanism for a pure spatiotemporal environmental response. ‘Turbulent coexistence’ is widely applicable to marine species with pelagic larval dispersal and relatively sessile adult life stages (and perhaps some wind-dispersed species) and complements other spatial and temporal storage effects previously documented for such species.

## Introduction

Simple ecological models predict that species competing for shared resources cannot coexist: the ‘superior competitor’ will eventually drive all other species to extinction. However, empirical exceptions to the competitive exclusion rule abound and substantial ecological theory has addressed the conditions that allow coexistence. In broad terms, the coexisting species must either have identical fitness (as in the neutral theory; Hubbell 2005) or differ in some ecological way ( ‘niche differences’ ) that reduces interspecific competition relative to intraspecific competition (Chesson 1991). These are two endpoints of a continuum (Adler *et al.* 2007), and in practice, communities of coexisting species exhibit processes that both reduce fitness differences ( ‘fitness-equalizing mechanisms’ ) and reduce the relative intensity of interspecific competition ( ‘stabilizing mechanisms’ ). The greater the fitness differences between species, the stronger the stabilizing mechanisms need to be and *vice versa* (Chesson 2000b). Stabilizing mechanisms can themselves be tradeoffs (e.g. stronger competitors are weaker dispersers; Tilman 1994) or ‘fluctuation-dependent mechanisms’ (e.g. the storage effect: Chesson 1984; Pacala & Tilman 1994) that require temporal or spatial variation in the environment (Chesson 1985) or endogenous fluctuations in density due to limit cycles or chaos (Armstrong & McGehee 1976; Holt & McPeek 1996).

Dispersal allows species to utilize space in different ways. When the environment is spatially heterogeneous, this can produce clear and intuitive niche separation, as each species preferentially disperses to, or performs best in, a particular environment type. Even if both species experience the environment in the same way, spatial heterogeneity in fitness can allow coexistence of species with differing proportions of dispersers, as conditions fluctuate between being favourable and unfavourable to dispersal (McPeek & Holt 1992; Holt & McPeek 1996). Even in a homogeneous environment, interspecific differences in average dispersal ability can promote coexistence. For example, a strict tradeoff between dispersal ability and competitive strength allows competitively inferior species to (temporarily) escape competition by colonizing patches newly cleared by disturbance (Tilman 1994). Competing parasitoids can coexist if spatial oviposition behaviour on host patches is sufficiently aggregated and they have either a tradeoff between competitive ability and reproductive fitness or a negative spatial correlation in oviposition behaviour (Klopfer & Ives 1997). Snyder & Chesson (2003, 2004) found that simple differences in the average dispersal distance could lead to coexistence via a storage effect mechanism. However, no studies have examined the role of dispersal *variability* in promoting coexistence.

High dispersal variability characterizes many marine organisms, especially those that live on rocky or coral reefs in nearshore waters. These organisms are relatively sessile as adults but release pelagic larvae whose dispersal is mediated by ocean currents (Kritzer & Sale 2006). Larvae are transported as they develop, settling and possibly recruiting to the adult stage at a new location if they reach suitable habitat. Many ecologically similar species often recruit to the same location and are able to coexist (Caselle & Warner 1996; Love *et al.* 1999). Communities that exhibit this life history include many coral reef fish, temperate fish collectively known as ‘rockfish’ (e.g. *Sebastes* spp.) and invertebrates such as sea urchins.

Individual larvae typically spend days to a few months in the pelagic phase (Moser & Boehlert 1991; Pasten *et al.* 2003) before settling within their ‘competency window.’ As these larvae are millimetres to centimetres in size, they have little influence on their horizontal motion, particularly in the early part of their pelagic phase, and are thus subject to the turbulent motions of the sea. The quasi-chaotic motions of the ocean mesoscale are typified by horizontal length scales of a few 10s of km and horizontal velocity scales of several 10s of km per day. Thus larvae once entrained into a coastal eddy can be advected several 10s to many 100s of km in the along-coast direction (e.g., Mitarai *et al.* 2008). Furthermore, larvae released within a few days of one another will follow similar paths as they are advected around coastal eddies (Mitarai *et al.* 2008; Siegel *et al.* 2008).

On the other hand, larval dispersal is often modelled as an advection–diffusion process (e.g. Jackson & Strathmann 1981). This assumes implicitly the simultaneous dispersion of many individual larvae, each with a path statistically independent from any other’s. This may be appropriate for assessing the long-term pattern of larval transport but will not describe transport for a single spawning season (Siegel *et al.* 2008). Most nearshore marine species have a brief spawning window of days to at most a few months, so the number of statistically independent dispersal paths emerging from a source location will be small (Siegel *et al.* 2003). Mitarai *et al.* (2008) implemented an ocean circulation model to simulate the flows that typify those off the west coast of North America. The simulated patterns of larval connectivity were spatially heterogeneous for a single spawning season and were highly variable among years. They also showed that the statistical properties of the connectivity patterns could be captured by caricaturing the process as a handful of successful dispersal events, where a single event links a contiguous group of source locations with a contiguous group of destination locations (Mitarai *et al.* 2008; Siegel *et al.* 2008). The number and size of these events, as well as the mean and variance of distance travelled by the larvae in an event, depend on the characteristics of the flow (such as mean eddy size), the length of the spawning season, and the duration of the pelagic dispersal period. Siegel *et al.* (2008) used these simulations of connectivity to help explain the extreme spatiotemporal variability usually observed in patterns of settlement (Dixon *et al.* 1999) and discussed the implications for fishery management.

In this study, we demonstrate that stochastic dispersal, as experienced by many nearshore marine organisms, can promote species coexistence. We analyse a simple competition model, loosely based on life history characteristics typical of shallow-dwelling reef fishes such as kelp rockfish (*Sebastes atrovirens*; Love *et al.* 2002). We have deliberately excluded priority effects, spatial or temporal heterogeneity in environmental quality, or life-history tradeoffs – while these processes may occur in rockfish, they are already known to promote coexistence, and we want to focus on the role of stochastic dispersal. Nevertheless, we find coexistence of two similar competing species (that could not coexist in a non-spatial model) if their spawning seasons do not perfectly overlap. We analyse this ‘turbulent coexistence’ with both spatially explicit simulations and a spatially implicit analytic model. We show that turbulent flow creates the opportunity for species to experience decorrelated settlement patterns, and that this decorrelation creates a spatial storage effect, in which the species are partitioning a ‘spatial niche.’ However, this spatial niche partition is ephemeral, fluctuating randomly through time and leaving no lasting spatial pattern – a result that is both conceptually novel and confounds easy biological intuition.

## Model Framework

We model two competing species distributed along a linear coastline, divided into evenly spaced sites of suitable habitat. Adults remain within a site and all competitive interactions are local. For simplicity, we abstract away many aspects of life history (age and size structure of adults, adult competition, spatial heterogeneity in habitat quality). Adult abundance in the population at site *x* in year *t +*1 depends on survival and new recruitment: 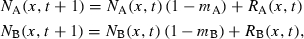
(1) where *N*_*i*_ and *R*_*i*_ are the adult and recruit abundances for species *i*, *m*_*i*_ is adult mortality and *x* and *t* index space and time. Competition occurs among settlers at a location, creating density-dependent recruitment: 
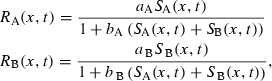
(2) where *S*_*i*_ is settler density and *a*_*i*_ and *b*_*i*_ are parameters in the Beverton-Holt recruitment function (widely used in fisheries modelling, there are theoretical reasons to expect this function will describe the outcome of competition among members of a cohort as they mature; White 2009). We assume that the inter- and intraspecific interaction strengths are the same for both species, because larvae of co-settling rockfish are often morphologically and ecologically indistinguishable (Wilson *et al.* 2008); this also makes coexistence particularly difficult. *S*_*i*_ (*x*, *t*) depends on the production and dispersal of larvae throughout the spatial domain: 
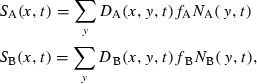
(3) where *D*_*i*_ (*x*,*y*, *t*) is the proportion of larvae produced by species *i* at location *y* that disperse to location *x* in year *t*, and *f*_*i*_ is the density-independent, per-capita production of larvae by adults of species *i*. Larval mortality during dispersal (from predation, starvation, and being swept out to sea) depends on the time in the plankton, which can span a range of days to months depending on the species; mortality variation due to the time required to disperse from *x* to *y* can be incorporated in *D*. The larval production term includes the average larval mortality and represents the expected number of settlers produced by an adult. Therefore, the expected value of 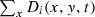 is equal to 1.

We allow the two competing species to differ in two ways. First, species A always has a higher per-capita productivity than species B ( *f*_A_*> f* _B_). Second, the species may have different dispersal kernels (*D*_*i*_ (*x,y,t* )) in any given year, reflecting differing patterns of turbulence during their spawning periods, but the statistical properties of the kernels are identical. All other parameters are identical between the two species, and are constant in space and time; hence, we drop the species-specific subscripts. While species A has a fitness advantage over species B, the two species are competitively equivalent: the relative frequency of recruits at a location is the same as the relative frequency of settlers. As the two species have identical average dispersal characteristics, species B has no advantage over species A, and there is no opportunity for coexistence via a tradeoff. Indeed, it is straightforward to show that, absent stochasticity or spatial heterogeneity, species A will always drive species B to extinction (unless *f*_A_ = *f* _B_– i.e., the two species are identical in every way), both in a non-spatial model and in a spatial model with diffusive dispersal.

In the single-species version of this model, *b* is a scaling parameter, controlling the carrying capacity given the values *f*, *a* and *m* : for a given equilibrium abundance *K*,
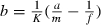
. The parameters *f*, *a* and *m* jointly control the intensity of density dependence when the population is at carrying capacity; in the analyses below we vary *f* to examine the effects of density dependence (higher fecundity produces more settlers, so that maintaining the same total recruitment at equilibrium requires more intense competition).

## Stochastic model of turbulent dispersal

We simulated larval dispersal in flow fields generated by the Regional Ocean Modelling System (Shchepetkin & McWilliams 2005; hereafter, the ‘ROMS model’ ), parameterized to broadly represent turbulent ocean flows off the California coast (Mitarai *et al.* 2008). We assumed that spawning lasts 30 days, and larvae can settle if they encounter the coastline between the ages of 20 and 40 days.

**Figure 1:**
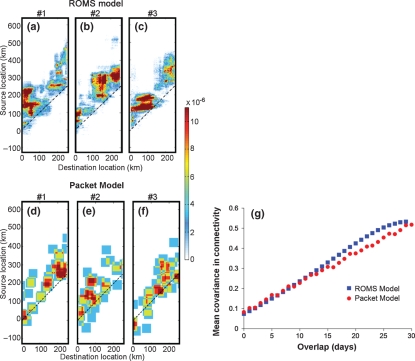
Simulations of realized dispersal in the ROMS model (a–c) and the packet model (d–f). Each of the three panels in each row represents a different year and the color scale represents the number of larvae dispersing from a given source to a given destination. (g) Correlation in connectivity patterns between the two species as a function of the overlap in their spawning windows; packet model results are means of 12 000 realizations and the ROMS results are means of 28 realizations. Parameter values for the dispersal models (used here and in rest of figures) are: *T*_sp_ = 30 days; *T*_L_ = 14 days; *r*=50 km; *C*=500 km. The resulting number of successful ‘packets’ per year (*P* ; eqn 4) is 21. The spatial variance in the packet model connectivity matrix is 0.5332, compared with 0.5362 for the ROMS model.

The connectivity patterns generated by the ROMS model are spatially heterogeneous and temporally stochastic; Fig. 1a–c shows three examples, revealing substantial interannual variability in connectivity. Larval release and settlement are spatially correlated, but the specific locations of these events change depending on the exact realization of the mesoscale (20–200 km) flow field (Mitarai *et al.* 2008; Siegel *et al.* 2008). These eddies collect larvae released from the nearshore over a large spatial scale and transport and deliver them as settlers in a cohesive unit. Averaged over many years, dispersal distance follows a normal distribution with a mean displacement of 135 km and a standard deviation (*σ*_*d*_ ) of 81 km. However, in any given year, connectivity is patchy and the patterns vary substantially from year to year.

The ROMS model runs too slowly to incorporate into a population model. Instead, we take advantage of previous work that developed a much simpler model that captures the general statistical patterns of larval dispersal in the turbulent ocean (Mitarai *et al.* 2008; Siegel *et al.* 2008), abstracting these statistical patterns into a ‘packet model’ by defining dispersal events ( ‘packets’ ) that link a group of source sites with a group of destination sites. The number of packets in a given year is 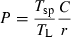
(4) (Siegel *et al.* 2008), where *T*_sp_ is the duration of spawning season, *T*_L_ is the characteristic time scale for settlement events, *C* is the length of the coastline and *r* is the scale of these settlement events (and is related to the size of eddying motions in the flow; see Siegel *et al.* (2008) for details). The destination location, *x*_*k*_, for the *k*th packet is selected randomly from within the domain while its source location, *y*_*k*_, is drawn from a normal distribution representing the long-term mean dispersal kernel. Connectivity matrices are then modelled based upon the number of packets between a given source and destination spread over the eddy scale, *r*: 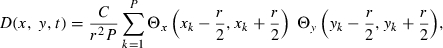
(5) where the boxcar function Θ_*x*_(*a*,*b* ) = 1 for *a*< *x*< *b* and zero otherwise, representing the destination and source area covered by each eddy (for integration with the spatially discretized population model, *r* /2 must be an integer multiple of the spacing between sites).

The packet model connectivity patterns are more artificial-looking than those from the ROMS simulations (Fig. 1d–f), but the spatial and temporal variances of *D*_*i*_ (*x,y,t* ) derived from the two models are similar, as are the spatial autocorrelation patterns (Figure S1). The patterns of variability within a year will also turn out to be important: how does realized connectivity vary between two seasons that do not overlap, or only partially overlap? For each site, we define an integrated measure of connection in a given year by summing *D* across all sources: 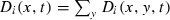. We calculated cov(*D*_*i*_ (*x,t* ), *D*_*i*_ (*x,t′*)), where *t*′ indicates a different spawning window within year *t*. This spatial covariance increases nearly linearly with the overlap in spawning seasons with similar patterns arising from the ROMS simulations and the packet model (Fig. 1g). Thus, the packet model provides a sound approximation to hydrodynamically realistic dispersal for use in spatially explicit population models.

## Spatially explicit simulation model

**Figure 2:**
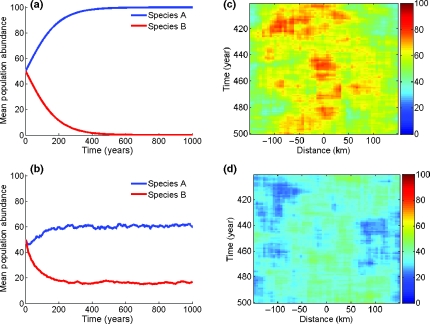
Left: Mean population size of both species through time using diffusive dispersal (a) and packet model dispersal with no overlap in spawning (b). For diffusive dispersal *σ*_*d*_ = 81 km. Right: Spatio-temporal patterns in population size using packet model dispersal for species A (c) and species B (d) for a 100 year time span and over the centre 300 km of the domain. Demographic parameter values (for this and other figures) are: *a*=1; *b*=0.045; *m*=0.1; *f*_A_ = 0.1818; *f*_B_ = 0.1727.

In the spatially explicit model with diffusive dispersal, the high-productivity species (species A) drives the low-productivity species (species B) to extinction (Fig. 2a). In contrast, the low-productivity species can persist when the two species disperse according to independent realizations of the packet model (Fig. 2b; assumes non-overlapping spawning seasons). Spatiotemporal patterns in adults of both species are patchy (Fig. 2c,d), with a weak tendency towards a negative correlation. Despite the spatial variability, mean abundances are relatively constant once the species approach their equilibria (Fig. 2b). Species A’s average abundance is reduced, relative to the deterministic model, by two processes: mean recruitment is reduced by nonlinear averaging across the variable number of settlers (accounting for *c.* 75% of the reduction) and competition with species B further reduces species A’s recruitment success.

**Figure 3:**
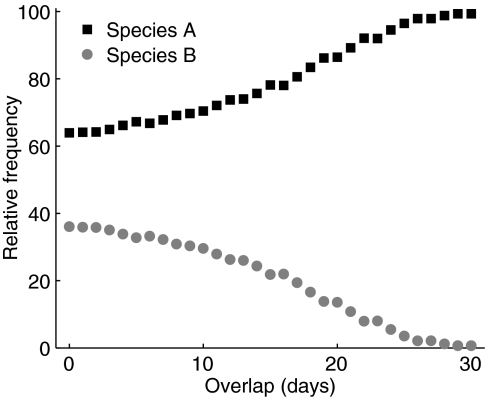
Mean percentage of the total population size for each species after 1000 years (mean of 50 simulations) over a range of overlap in spawning from none to complete.

Coexistence in this model requires some decorrelation in settlement: the two species must not have exactly the same realized dispersal kernel, which would arise if they had exactly the same spawning season and competency window. Coexistence depends on the overlap in spawning seasons (Fig. 3). As the amount of overlap increases, the equilibrium abundance of species B declines until it is effectively extinct, above an overlap of 25 days. For this example, coexistence requires that the correlation between the dispersal kernels is < 80%.

This model contains several processes and emergent patterns that might contribute to coexistence. For example, larval settlement is highly aggregated (i.e. more clumped than spatially random, with a variance greater than the mean); this might promote coexistence through mechanisms such as those found in host–parasitoid models (Klopfer & Ives 1997). This aggregation, together with the spatial autocorrelation in settlement incorporated in the packet model, leads to substantial spatio-temporal patterning in adult abundance (Fig. 2). If average dispersal distances are short relative to the scale of adult pattern, then coexistence might arise because settlement primarily occurs in conspecific patches (Snyder & Chesson 2003). Finally, the combination of intraspecific variability and imperfect interspecific correlation in settlement suggests that a storage effect may be acting. To tease these apart we turn to a simpler model, containing only the latter mechanism.

## Spatially implicit model

To focus on the role of dispersal variability in promoting coexistence, we develop a spatially implicit model that strips away the potentially confounding factors discussed above. First, we eliminate intra- and interspecific patterns in adult density, forcing adult density to be homogeneous at the end of each time step: 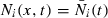. This is not meant to be a biologically realistic approximation (although it could be achieved by assuming a high rate of adult movement); rather, we are artificially intervening to ensure that any remaining coexistence is not due to adult spatial patterning.

Second, we eliminate the aggregation and spatial autocorrelation in settlement, focusing on the simple effects of spatial variances (regardless of magnitude) and interspecific correlations. Because adult densities are homogeneous, the number of settlers at location *x* is 
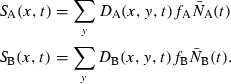
(6) As the sum of the dispersal kernels over all sources have an expected value of 1, the expected number of settlers depends only on *f*_*i*_ and *N*_*i*_ (*x*, *t*): 
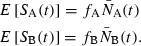
(7) The variability in settler numbers depends on the statistics of *D*_*i*_ (*x*,*y*, *t*): 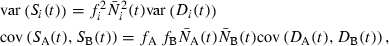
(8) where the variances (which can be arbitrarily small) and covariance are across space. We do not incorporate spatial autocorrelation, and we assume that all higher moments of dispersal variation are zero.

As species A has higher fitness, the coexistence criterion is that species B must be able to increase when it is at low density (Chesson 1994). Species B’s growth rate when rare is: 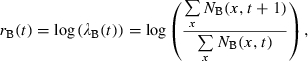
(9) where *N*_B_(*x*,*t*) is small for all *x* and *N*_A_(*x*, *t*) is at its single-species equilibrium. Coexistence requires that *E*[*r*_B_(*t*)] > 0. For simplicity of exposition, we assume that the temporal fluctuations in *λ*_B_(*t*) are small, so an approximate coexistence criterion is *E*[*λ*_*B*_(*t*)] > 1. This means that average per-capita recruitment must exceed adult mortality: 
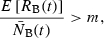
(10) where *E*[*R*_B_(*t*)] is the expected number of recruits at time *t* and 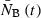 is the spatial mean adult population. As species A is at equilibrium, its expected per-capita recruitment equals its mortality: 
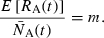
(11) As species B is at low density, the per-settler recruitment rate primarily depends on the number of species A’s settlers: 
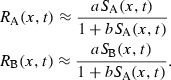
(12) We now estimate the expected recruitment of both species, keeping terms up to second order in the Taylor expansion around the mean number of settlers: 
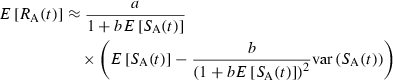
(13)
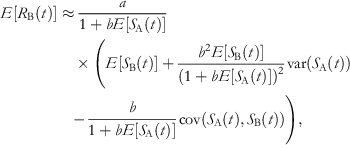
(14) where expectations, variances and covariances are over space.

We set species A is to its single-species stochastic equilibrium, K*, and species B to a low density, *B*_0_. Thus, 
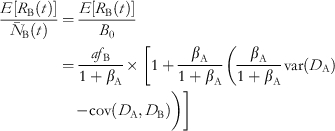
(15) (where *β*_A_ = *b f*_A_K* is related to the strength of density dependence at equilibrium).

From the equilibrium conditions for species A, 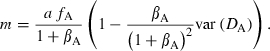
(16) Substituting eqns 15 and 16 into inequality 10 gives the coexistence condition in terms of the ratio of productivity between the species: 
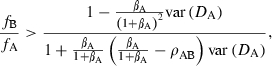
(17) where *ρ*_AB_ is the correlation in the connectivity patterns between the two species. This can be evaluated using estimates of the dispersal variance and covariance from the packet model.

**Figure 4:**
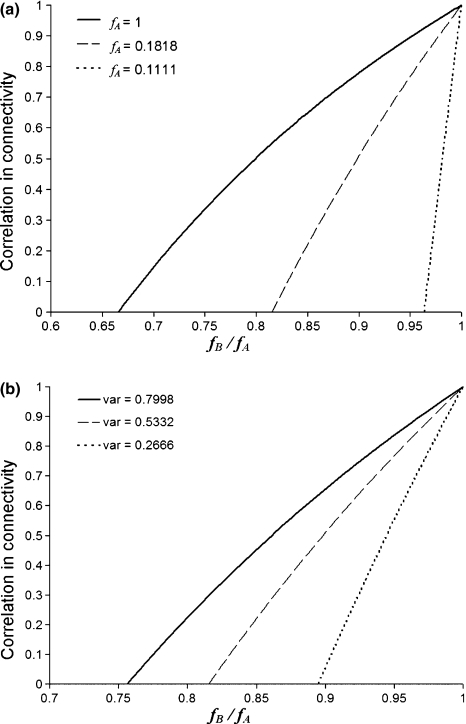
Coexistence thresholds from the spatially implicit model, relating correlation in settlement and the fecundity ratio of species B to species A. Coexistence occurs to the right of the lines. Panel (a) varies the intensity of density dependence by changing the fecundity of Species A. Panel (b) varies the variance in settlement.

The higher the correlation in dispersal, the more demographically similar the species need to be in order to coexist (Fig. 4). The range of fitness differences over which coexistence is possible expands with increasing intensity of competition ( *f*_A_; Fig. 4a) and increasing variance in connectivity (Fig. 4b). There is no limiting similarity in this model: coexisting species can have arbitrarily small differences in dispersal patterns, as long as the demographic differences are also small. A somewhat counterintuitive result is that the reduced mean abundance of the resident under stochastic dispersal (compare Figs 2a and 2b), which might be expected to reduce the intensity of competition and thereby make coexistence easier, instead reduces *β*_A_ and thereby makes coexistence more difficult. This can be understood by recognizing that the variance in settler abundance is proportional to the square of mean adult abundance, so reducing the mean adult abundance disproportionally reduces the spatial variability in the competitive environment available for the invading species to exploit.

## Synthesis of spatially explicit and spatially implicit models

**Figure 5:**
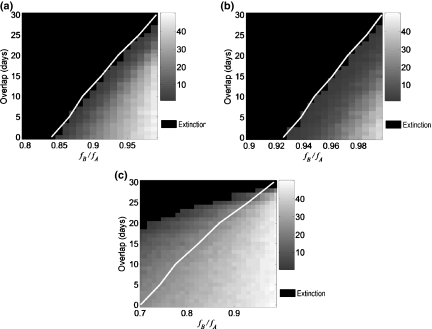
Coexistence thresholds estimated from the spatially explicit and spatially implicit models. (a) All parameters as in Figure 2. (b) *f*_A_ = 0.1290. (c) *f*_A_ = 1. The white line indicates the coexistence threshold from the spatially implicit model; coexistence is predicted to the right of the line. Grey indicates the proportion of species B in the population after 1000 years in the spatially explicit model (averaged over five simulations at each parameter combination). Black indicates that species B is ‘extinct’ (the simulation model does not allow absolute extinction, so we define competitive exclusion to have occurred if species B is below 1% of the total population after 1000 years).

Does the spatially implicit model, with its reduced set of features and processes, capture the coexistence properties of the spatially explicit model? To answer this question, we simulated the latter model over a range of productivity ratios (fitness inferiority of species B) days of spawning overlap (correlation in dispersal) and values of species A’s productivity (intensity of competition). Except when the density dependence is strongest, the spatially implicit model predicts coexistence in the spatially explicit model almost perfectly (Fig. 5a,b). This provides strong evidence that coexistence in the spatially explicit model is predominantly produced by spatial variability in settlement combined with some level of settlement decorrelation between species; turbulent dispersal is simply providing a means to achieve appropriate settlement statistics. The other phenomena in the simulation model (spatial patterns in adult density, aggregation in settlement) are quantitatively and qualitatively irrelevant to coexistence.

**Figure 6:**
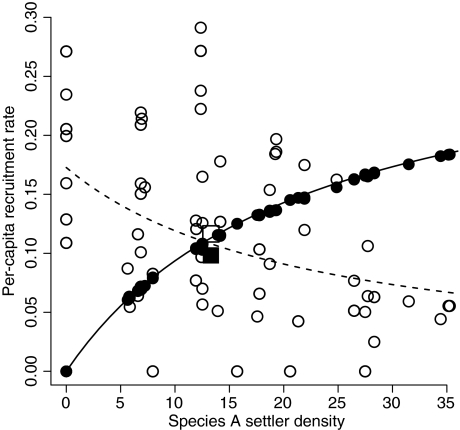
The per-capita recruitment rate of species A (solid symbols and curve) and species B (open symbols and dashed curve), as a function of species A settler density, when species B is rare. This is a snapshot in time, with the variation being across space. The circles represent each patch in a simulation of the spatially explicit model, and the curves are the predicted values of actual (species A) and expected (species B) recruitment rates in the spatially implicit model (see Appendix S1). The squares mark the mean settler density and recruitment rates in the spatially explicit model, revealing that the nonlinearities in the recruitment curves cause settlement variability to reduce the mean recruitment rate of species A and increase the mean recruitment rate of species B. All parameters as in Fig. 2.

We can understand how this coexistence mechanism operates by examining per-capita recruitment rates when species B is at low density (Fig. 6). For species A, this relationship follows the Beverton-Holt curve, with a negative second derivative, and spatial variability in settlement reduces the mean recruitment rate. In contrast, if species B has an independent spawning season, then its expected recruitment rate is inversely proportional to the density of resident settlers, with positive second derivative, and settlement variability increases its mean recruitment rate. Effectively, the advantage that species B enjoys in sites where resident settlement is low outweighs the disadvantage that it suffers where resident settlement is high. As the correlation in settlement increases, species B is less likely to have high settlement rates in patches where A’s settlement is low and loses much of that relative advantage (Figure S2; Appendix S1).

In the spatially implicit model, increasing the intensity of competition increases the nonlinearity of the recruitment rate curves, in particular giving species B a stronger recruitment advantage in patches with little resident settlement (Figure S3). However, this is insufficient to explain the increased coexistence region under strong competition in the spatially explicit model (Fig. 5c). The latter discrepancy may simply reflect a failure of our approximations (using *E*[*λ*_B_(*t*)] instead of *E*[*r*_B_(*t*)], disregarding higher moments of dispersal variability). Alternatively, with large *f* even a single settlement event suffices to saturate the recruitment function, so that the discreteness and spatiotemporal correlation of settlement patterns associated with turbulent flow (and not merely their variances) may become important.

## Turbulent coexistence is a spatial storage effect

Turbulent coexistence is not a simple tradeoff, for there is no circumstance in which species B has a direct fitness advantage over species A. It is a fluctuation dependent mechanism because coexistence is impossible when the variance in dispersal goes to zero, in which case the right hand side of inequality 17 becomes one. Although adult mortality affects the coexistence criterion (through *β*_A_), coexistence is possible if *m*=1, and thus it cannot be a temporal coexistence mechanism (storage effect or relative nonlinearity of competition).

Here, we demonstrate that turbulent coexistence is, at least predominantly, a spatial storage effect. A key component of this mechanism is that the spatial covariance between the ‘environmental response’ (density-independent growth rate in each patch, *E*_*x*_) and the ‘competitive response’ (reduction in growth rate due to inter- and intraspecific interactions, *C*_*x*_) should be weaker for the rare species than for the common species (Chesson 2000a; Sears & Chesson 2007). As the spatial variation in population growth is only due to recruitment variation, the environmental and competitive responses can be represented by the log of the per-capita settlement rate and the log of the ratio of settlers to recruits, respectively (Chesson 1997): 
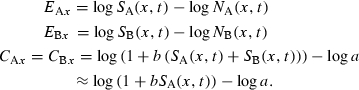
(18) In the spatially implicit model, the adult densities are the same in all patches, so it is evident that 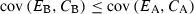, with equality only if *ρ*_AB_ = 1. In the spatially explicit model with the parameter values from Fig. 2 and no overlap in spawning seasons, the covariances actually have opposite signs (Figure S4).

## Discussion

We have demonstrated a novel coexistence mechanism, ‘turbulent coexistence’, in which stochastic dispersal in a spatially structured population can allow a less productive species to coexist with a more productive one. Stochastic dispersal is the driver of this coexistence mechanism: in contrast to other models, coexistence does not depend on the longevity of the species (and is possible with non-overlapping generations), temporal fluctuations in any environmental conditions other than those controlling dispersal, local vs. long-distance dispersal, spatially heterogeneous post-settlement environments or priority effects. Unlike other spatial coexistence mechanisms, it does not require exogenous variability in the environmental conditions that influence birth and death rates (Chesson & Warner 1981), heterogeneity in habitat quality or preference (Snyder *et al.* 2005), resource partitioning (Brown *et al.* 1997) or among-species differences in dispersal ability (Snyder & Chesson 2003, 2004). All that is required is that the two species make differential use of the turbulent flow so that they can have imperfectly correlated patterns of connectivity. These patterns of connectivity are, indeed, driven by variability in the physical environment (notably the wind fields that determine the realized mesoscale flow patterns), but coexistence theory has not previously examined the implications of environmental variability affecting dispersal. Coexistence becomes easier as the correlation between connectivity patterns decreases or as the spatial variance in connectivity increases; aggregated settlement patterns such as those seen in the packet model promote coexistence by generating high spatial variance, but are not intrinsically necessary.

The spatial storage effect can be viewed as a partitioning of a ‘spatial niche’ (Chesson 2000a). In our models, this involves interspecific differences in the spatial distribution of settlers. The spatially implicit model reveals that, mathematically, the only requirement for coexistence to be feasible is spatially variable settlement that is not perfectly correlated between species. The ROMS and spatially explicit simulation models demonstrate that turbulent larval transport allows species to partition this spatial niche by spawning at different times. Spawning time is not itself a niche axis; rather, it is the biological difference that allows the species to partition the niche, just as differences in gape size allow coexisting consumers to partition a niche axis of prey size. Indeed, other biological differences might allow species to exploit turbulence to generate decorrelated dispersal patterns (Mitarai *et al.* 2008; Siegel *et al.* 2008) including different lengths of dispersal periods (Kinlan & Gaines 2003) and different behaviour of larvae (e.g. vertical or horizontal migration) during dispersal (Siegel *et al.* 2008).

Turbulent coexistence is qualitatively similar to the spatial lottery model (Chesson 2000a), although in that model the competitive response depends on the local density of adults as well as settlers, and a mechanism for spatio-temporal variability in settlement was not discussed. In both models, settlement variability as the environmental response represents ‘pure spatiotemporal environmental variation’ in the language of Chesson (1985); our work is the first demonstration of a physical mechanism generating such variation through spatiotemporal dispersal variability.

Subsequent models of the spatial storage effect, moreover, focused on spatial heterogeneity in the environmental response that does not vary in time ( ‘pure spatial environmental variation’ ), representing either site conditions that affect post-settlement demography (Chesson *et al.* 2005; Sears & Chesson 2007) or post-settlement competitive ability. The chaotic coexistence models of Holt & McPeek (1996; see also related work by McPeek & Holt 1992) also require spatial heterogeneity in carrying capacity; they produce spatial niche partitions, as the strong dispersers are predominantly in the low-*K* patch and *vice versa*. Such persistent partitions of the spatial niche can easily be visualized; in contrast, our model generates ephemeral niche partitions that vary stochastically from year to year, leaving no persistent pattern on the landscape.

The lack of a tradeoff distinguishes our results from a broad class of spatial coexistence mechanisms, such as Tilman’s (1994) competition-dispersal tradeoff and Klopfer & Ives (1997) parasitoid model (the latter model allows coexistence without a tradeoff only if the oviposition patterns are negatively correlated between species). The biological communities that motivated our work almost certainly contain tradeoffs; but they are not necessary for the turbulent coexistence mechanism, and, as their effects are well understood, we have chosen not to focus on them.

We have stressed turbulence-driven stochastic variability in dispersal as the source of the settlement decorrelation required for coexistence. However, deterministic differences in dispersal patterns (e.g. through differences in mean currents between seasons) may also produce the necessary spatial decorrelation in settlement. For example, in the Southern California Bight, winter-spawned larvae tend to be transported poleward along the mainland coast while summer-spawned larvae are more likely advected equatorward (Mitarai *et al.* 2009). This produces a persistent partition of the spatial niche, and will likely create spatial decorrelation of adult abundances as well (S. Mitarai, unpublished results). Because seasonal variability and eddy time scale variability are comparable in magnitude (Mitarai *et al.* 2009), the differences in spawning season required are far larger than under the turbulent coexistence mechanism that we have focused on.

Rockfish in the genus *Sebastes* form remarkably diverse communities along the west coast of North America (Love *et al.* 2002), and their general life histories – long-lived, sedentary adults (Love *et al.* 2002) with annual mortality (*m*) typically 0.05–0.15 (Cailliet *et al.* 2000), pelagic larvae with variable spatiotemporal settlement patterns (Wilson *et al.* 2008) and intense competition among settlers (Johnson 2006a,b, 2007) – parallels our model. Both peak spawning periods and pelagic larval durations are 1–2 months for many species (Moser & Boehlert 1991; Cailliet *et al.* 2000). While adults generally segregate across subtle differences in bottom habitat, newly settled juveniles often occur in multispecies groups (Ammann 2004) and probably experience interspecific competition at that stage. Although we do not claim that our model provides the sole coexistence mechanism for these species, we suggest that it can contribute strongly. Particularly interesting are two ‘species complexes’ described by Wilson *et al.* (2008)– groups of three and four species, respectively, that share very similar morphology, habitat preferences, life histories and spawning seasons. Based on our model, we would predict that, within a complex, the species show differing spatio-temporal patterns of settlement. Spawning seasons have not been precisely delineated for most species, but gopher and kelp rockfish (in the KCGB complex) have different peak spawning months (February–March and May, respectively; Love *et al.* 2002). Unfortunately, field identification of settling juveniles within a complex is impossible. However, work is ongoing to use genetic markers to identify archived samples (J.E. Caselle, personal communication), which will allow these ideas to be tested.

Density-dependence in many rockfish species primarily involves juvenile density (Love *et al.* 2002; Hart & Sissenwine 2009), but in other species recruitment may also be affected by local adult density, which can promote coexistence through temporal storage effects such as the lottery model (Chesson & Warner 1981). Such mechanisms require variability in adult density, and so would not operate in our spatially implicit model. However, spatially uniform adult density-dependence reduces the intercept of the per-settler recruitment function (parameter *a* in Eq. (2)). This reduces the intensity of larval competition and thereby increases the settlement decorrelation that is required for coexistence. In other words, the spatially uniform adult density effects smooth out the spatial heterogeneity in the competitive environment, making a spatial storage effect more difficult. Spatially explicit simulations, using a model of combined settler and adult density effects from White (2009), generate variation in adult density, but reveal similar results: coexistence is still possible, but a greater difference in spawning seasons is required for given set of demographic rates. Our models produce little to no spatial correlation between settler and adult abundances; but in systems with strong local recruitment, adult density-dependence might qualitatively change the coexistence criteria.

Mathematically, a key component of the coexistence mechanism is the function multiplying var(*S*_A_) in eqn 13, which is proportional to the second derivative of *R*/*S* (the per-settler recruitment rate), evaluated at the expected number of resident settlers. We expect that any density dependence function with positive second derivative will allow coexistence via this mechanism. Any recruitment function must eventually have positive second derivative, as it asymptotes to zero. However, if the function is sigmoid, with mean resident settler densities in the region of negative second derivative, then turbulent dispersal would not promote coexistence.

This theory may also apply to non-marine systems, such as wind-dispersed plants and invertebrates. Most wind-dispersed seeds do not leave the convective boundary layer and are dispersed for only a few minutes. Averaged across the fruiting season, the dispersal kernel will not be stochastic, just as in marine species with pelagic durations of a few hours (Mitarai *et al.* 2008), although the kernel may be very different from Gaussian (Kuparinen 2006). However, small seeds and some invertebrates with sufficient buoyancy/lift to leave the boundary layer may disperse for weeks, similar to Saharan dust clouds, and will likely experience an eddy-driven collecting and deposition process (Drake & Farrow 1989) that is analogous to the turbulent ocean circulation simulations presented here.

Finally, we must ask whether this coexistence mechanism extends beyond two-species communities. We performed three-species simulations of the spatially explicit model and found that three-species coexistence is possible, even if one of the species has a spawning season that always overlaps with one or the other of its competitors. The details of the coexistence criteria appear complex: in some scenarios, reducing the fitness differences between species leads to exclusion, suggesting a form of limiting similarity, while in other scenarios this enhances coexistence (Appendix S2). This would be a fruitful direction for further analysis.

## SUPPORTING INFORMATION

**Figure S1** Spatial autocorrelation in connectivity for the two dispersal models, measured from the perspective of settlement sites.

**Figure S2** Per-capita recruitment for both species over a range of the number of settling larvae of species A, for three levels of correlation in dispersal.

**Figure S3** Effects of the intensity of competition on per-capita recruitment when species B is rare and settlement is uncorrelated.

**Figure S4** Spatial correlations between the environmental response (*E_x_*) and the competitive response (*C_x_*) when species B is at low density.

**Appendix S1** Recruitment rates in the spatially implicit model.

**Appendix S2** Simulations of three-species coexistence.

As a service to our authors and readers, this journal provides supporting information supplied by the authors. Such materials are peer-reviewed and may be re-organized for online delivery, but are not copy-edited or typeset. Technical support issues arising from supporting information (other than missing files) should be addressed to the authors.
