# Supplementary material for: Turbulent dispersal promotes species coexistence
Source: Ecol Lett. 2010 Mar;13(3):360–71. doi: 10.1111/j.1461-0248.2009.01427.x (PMC2847191; doi:10.1111/j.1461-0248.2009.01427.x)

Figure S1: Spatial autocorrelation in connectivity for the two dispersal models, measured from the perspective of settlement sites. Based on 28 replicate ROMS simulations and 9000 packet model realizations.


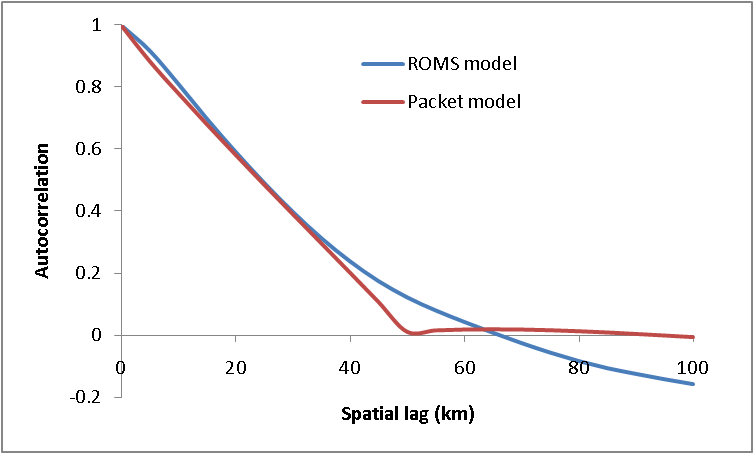

Supplement: Supplementary file 1 [file ele0013-0360-SD1.doc]
