# Supplementary material for: Turbulent dispersal promotes species coexistence
Source: Ecol Lett. 2010 Mar;13(3):360–71. doi: 10.1111/j.1461-0248.2009.01427.x (PMC2847191; doi:10.1111/j.1461-0248.2009.01427.x)

Figure S2: Per-capita recruitment for both species over a range of the number of settling larvae of species A, for three levels of correlation in dispersal. These are derived from the spatially implicit model, using formulas described in Appendix S1. The curve for species B is expected recruitment. Parameters used: *a* = 1, b = 0.09, *f_A_* = 0.5, *f_B_* = 0.45, *K_A_* = 100.


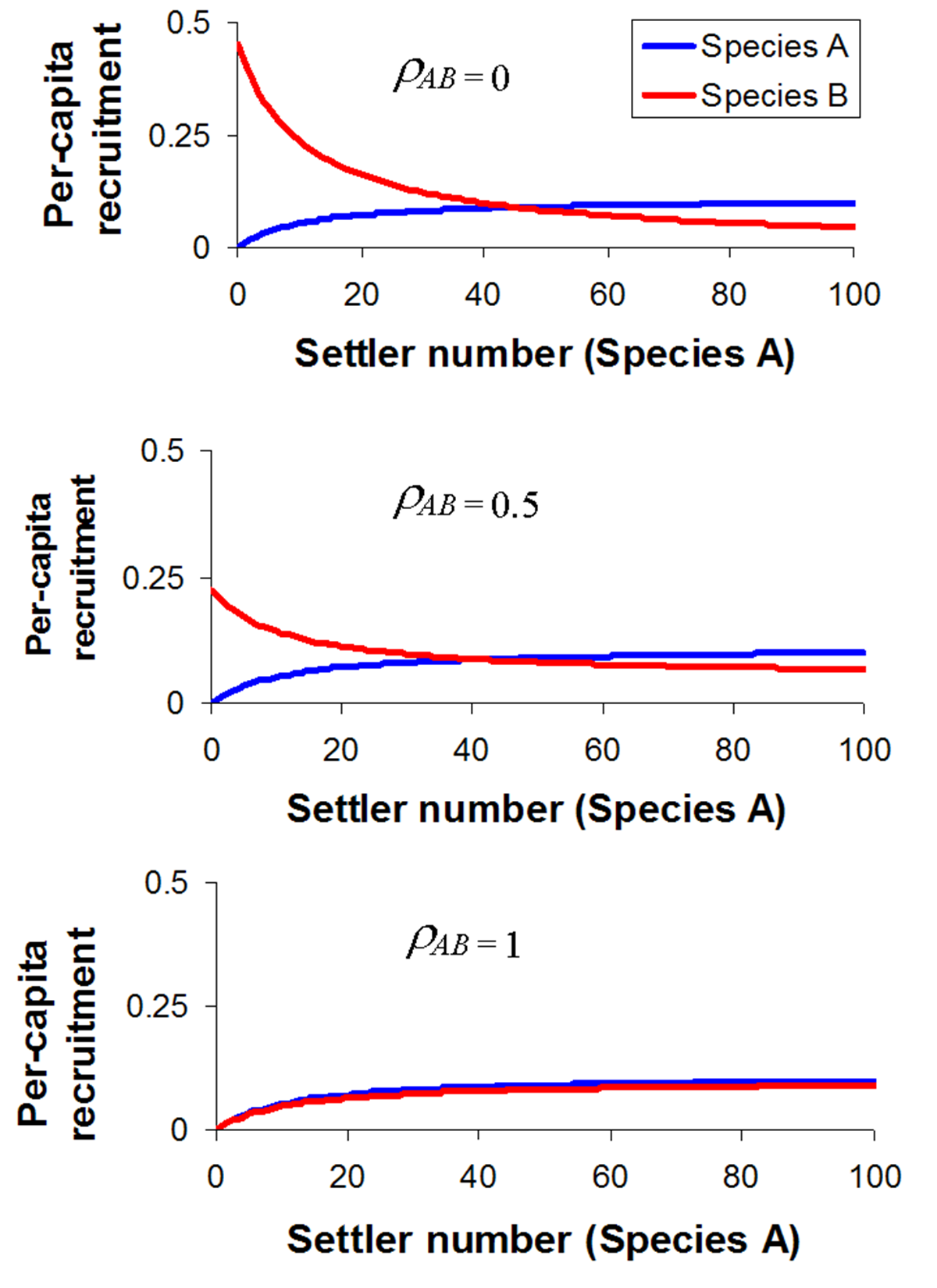

Supplement: Supplementary file 2 [file ele0013-0360-SD2.doc]
