# Supplementary material for: Turbulent dispersal promotes species coexistence
Source: Ecol Lett. 2010 Mar;13(3):360–71. doi: 10.1111/j.1461-0248.2009.01427.x (PMC2847191; doi:10.1111/j.1461-0248.2009.01427.x)

Figure S3: Effects of the intensity of competition on per-capita recruitment when species B is rare and settlement is uncorrelated. (A) Baseline parameters (mean number of settlers ~13); (B) parameters as in Figure 5C (mean number of settlers ~70). The curve for species A is actual per-capita recruitment rate, while for species B it is expected per-capita recruitment. Species B’s relative advantage in sites with few settlers of species A is greatly enhanced by the increased competition intensity


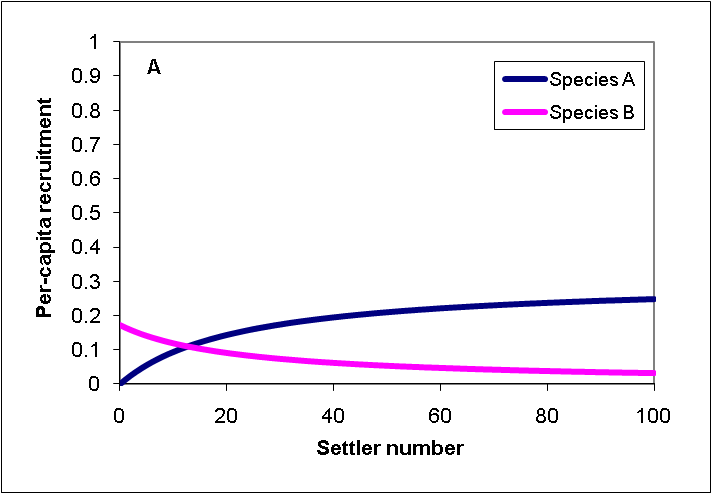


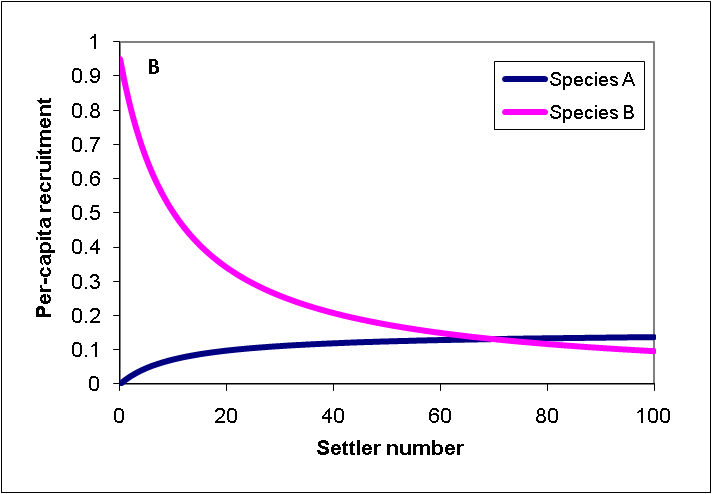

Supplement: Supplementary file 3 [file ele0013-0360-SD3.doc]
