# Supplementary material for: Turbulent dispersal promotes species coexistence
Source: Ecol Lett. 2010 Mar;13(3):360–71. doi: 10.1111/j.1461-0248.2009.01427.x (PMC2847191; doi:10.1111/j.1461-0248.2009.01427.x)

Figure S4: Spatial correlations between the environmental response (*E_x_*) and the competitive response (*C_x_*) when species B is at low density. Species A (resident) is red; species B (invader) is blue; each point represents the conditions in a particular site, all evaluated at one time. Parameters as in Figure 2, with no overlap in spawning seasons; species A was run by itself for 900 years to reach equilibrium, then species B was introduced at very low density and allowed to increase for 100 years, at which point it was about 4% of the total population.


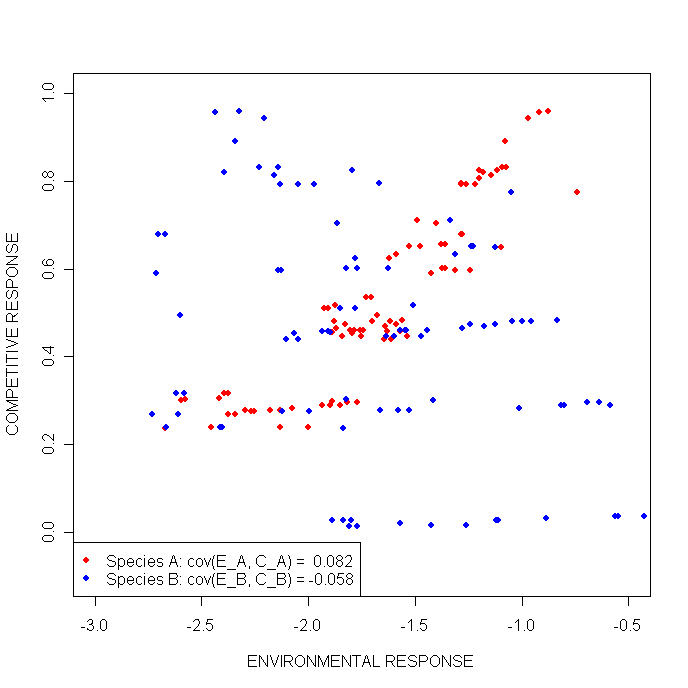

Supplement: Supplementary file 4 [file ele0013-0360-SD4.doc]
