# Supplementary material for: Turbulent dispersal promotes species coexistence
Source: Ecol Lett. 2010 Mar;13(3):360–71. doi: 10.1111/j.1461-0248.2009.01427.x (PMC2847191; doi:10.1111/j.1461-0248.2009.01427.x)
Supplement: Supplementary file 5 [file ele0013-0360-SD5.doc]

# Appendix S1: Recruitment rates in the spatially implicit model

The key component for coexistence is the density dependent per-capita recruitment rate, *R*/*N*. The local recruitment rate of species A is:

| $\frac{R_{A}(S_{A})}{K}=\frac{1}{K}\frac{aS_{A}}{1+bS_{A}}$, | (S1) |
| --- | --- |

where the recruitment of species A depends only on the settlement of species A. For species B, the recruitment of species B depends on the settlement of both species. So the local per-capita recruitment rate is:

| $\frac{R_{B}(S_{A},S_{B})}{B_{0}}=\frac{1}{B_{0}}\frac{aS_{B}}{1+bS_{A}}$. | (S2) |
| --- | --- |

Now, the expected per-capita recruitment rate is conditional on the settlement of species A:

| . | (S3) |
| --- | --- |

Thus, we need to solve for $E\left[S_{B}\left|S_{A}\right.\right]$:

| . | (S4) |
| --- | --- |

To solve for the second term in Equation (S4) we used Equations (15):

| , | (S5) |
| --- | --- |

which becomes:

| , | (S6) |
| --- | --- |

where $\rho_{AB}$ is the correlation in the dispersal of both species.

Then, we substitute Equations (14) and (S6) into Equation (S4) to get:

| , | (S7) |
| --- | --- |

which simplifies to:

| . | (S8) |
| --- | --- |

If we assume the variance in dispersal is the same for both species, which would be true if the spawning periods are the same, then Equation (S8) simplifies to:

| . | (S9) |
| --- | --- |

Then, we substitute Equation (S9) Into Equation (S3) To get the expected per-capita recruitment rate of species B conditional on the settlement of species A:

| . | (S10) |
| --- | --- |
| Equations (S1) and (S10) are used to generate curves in Figures 6, S2, and S3. |  |
