# Supplementary material for: Turbulent dispersal promotes species coexistence
Source: Ecol Lett. 2010 Mar;13(3):360–71. doi: 10.1111/j.1461-0248.2009.01427.x (PMC2847191; doi:10.1111/j.1461-0248.2009.01427.x)
Supplement: Supplementary file 6 [file ele0013-0360-SD6.doc]

# Appendix S2: Simulations of three-species coexistence

We modified the spatially explicit model to include three species: species A spawns first, followed by species B, and finally species C. We modeled three levels of overlap in spawning season: none; 10 days (33%) overlap between adjacent species; and 20 days (67%) overlap between adjacent species. In the latter scenario species B had no dispersal that was fully independent of the other species. We modeled two rankings of productivity: *f_A_* > *f_B_* > *f_C_* and *f_B_* > *f_A_* > *f_C_*. Finally, we examined two sets of productivity ratios: *f*_med_/*f*_high_ = 0.95 and *f*_low_/*f*_high_ = 0.90; and *f*_med_/*f*_high_ = 0.975 and *f*_low_/*f*_high_ = 0.95. All other parameters are as in Figure 2. At 20 day overlap, the two-species model predicts that species with a productivity ratio of 0.95 should be able to coexist but those with a ratio of 0.9 should not; at 10 and zero day overlaps, all pairs of species should be able to coexist (Figure 5A).

When species A has the highest productivity, all three species coexist across all overlap scenarios with the wide productivity ratios, but species B goes extinct at the narrow productivity ratio and the widest overlap (Figures S2.1 – S2.3). This result (exclusion when the demographic fitnesses are more similar) is opposite to the result from the two species model, and suggests limiting similarity (if species A and C are too similar, then there is no room for an intermediate species B to persist).

In contrast, when species B has the highest productivity, then species C goes extinct at wide productivity ratios but not at narrow productivity ratios, for both 10 and 20-day overlap scenarios (Figures S2.4 – S2.5). At 20 day overlap this is expected, as exclusion should occur when *f_C_ / f_B_* = 0.9; but it is unexpected when the spawning overlap is only 10 days.


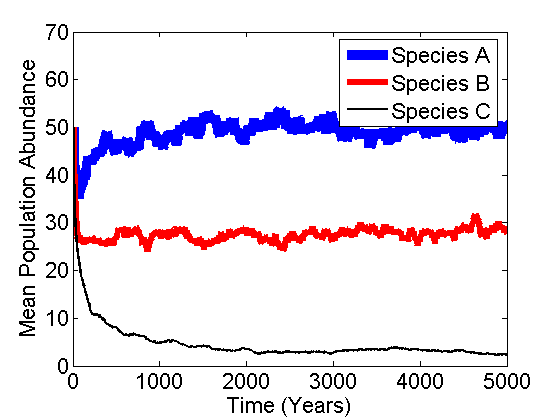

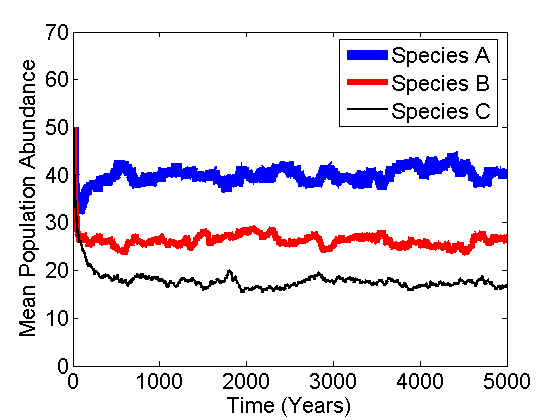


Figure S2.1: No overlap in spawning seasons. Left panel: *f_A_* = 0.1818; *f_B_* = 0.1727; *f_C_* = 0.1636. Right panel: *f_A_* = 0.1818; *f_B_* = 0.1763; *f_C_* = 0.1727. Long-term coexistence seems likely on left, and certain on right.


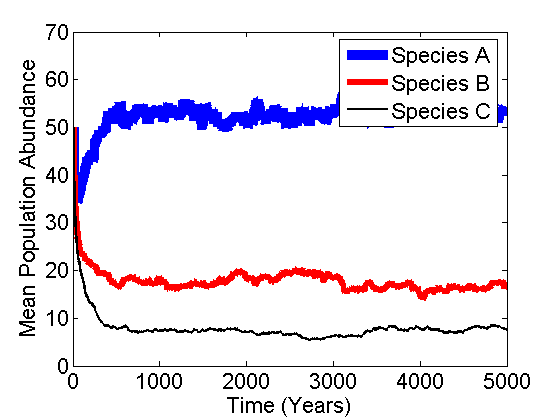


Figure S2.2: Spawning seasons, in days: species A 1-30, species B 21-50, species C 41-70. Thus all species have some days when they are spawning alone. *f_A_* = 0.1818; *f_B_* = 0.1727; *f_C_* = 0.1636


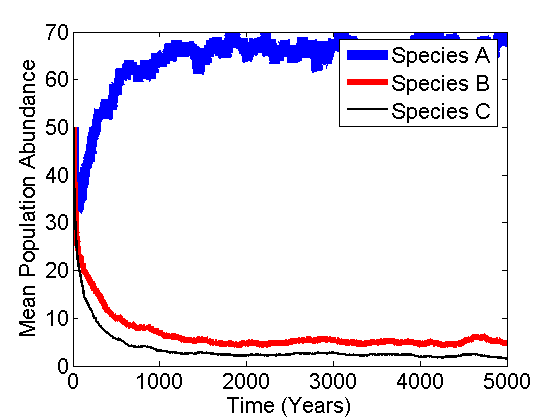

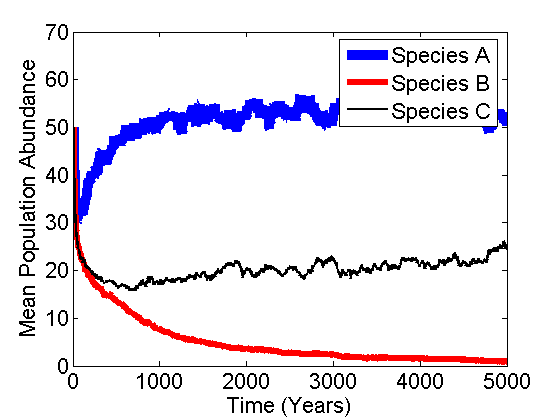


Figure S2.3: Spawning seasons, in days: species A 1-30, species B 11-40, species C 21-50. Thus species B is never spawning by itself. Left panel: *f_A_* = 0.1818; *f_B_* = 0.1727; *f_C_* = 0.1636. Right panel: *f_A_* = 0.1818; *f_B_* = 0.1763; *f_C_* = 0.1727. Long-term coexistence seems likely on left, but species B is heading to extinction on right.


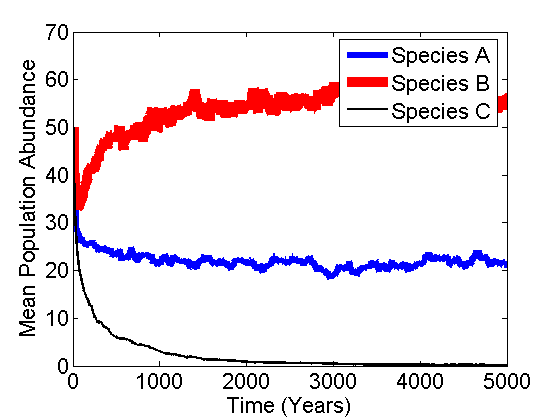

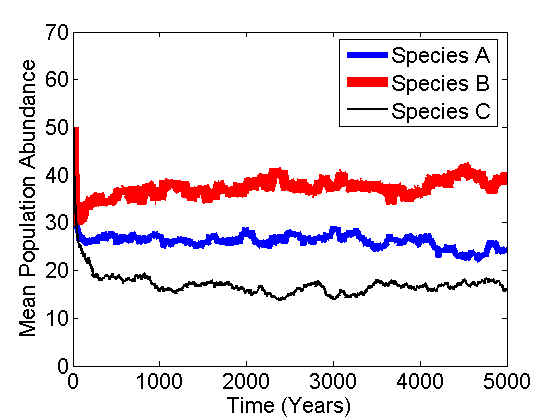


Figure S2.4: Spawning seasons as in Figure S2.2, but species B has the highest fecundity. Left panel: *f_A_* = 0.1727; *f_B_* = 0.1818; *f_C_* = 0.1636. Right panel: *f_A_* = 0.1763; *f_B_* = 0.1818; *f_C_* = 0.1727. Species C goes extinct on left, but persists on right.


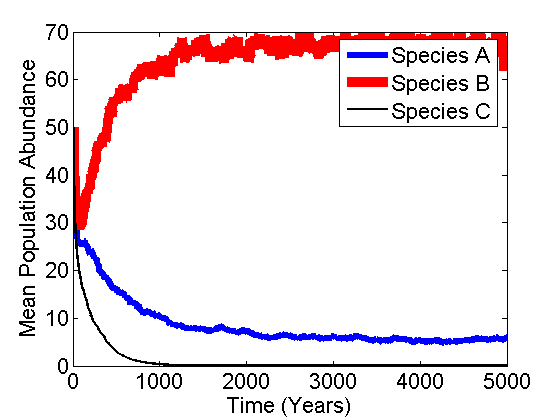

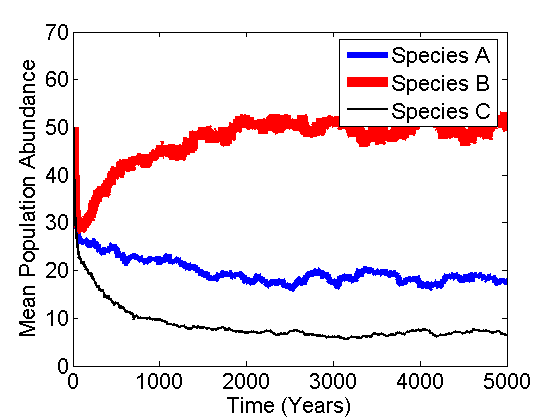


Figure S2.5: Spawning seasons as in Figure S2.4, but species B has the highest fecundity. Left panel: *f_A_* = 0.1727; *f_B_* = 0.1818; *f_C_* = 0.1636. Right panel: *f_A_* = 0.1763; *f_B_* = 0.1818; *f_C_* = 0.1727. Species C goes extinct on left, but persists on right.
